# Keeping up with the genomes: scaling genomic variant interpretation

**DOI:** 10.1186/s13073-019-0700-4

**Published:** 2019-12-31

**Authors:** Heidi L. Rehm, Douglas M. Fowler

**Affiliations:** 1 Center for Genomic Medicine, Massachusetts General Hospital, Cambridge Street, Boston, MA 02114 USA; 2 Medical and Population Genetics, Broad Institute of MIT and Harvard, Main Street, Cambridge, MA 02142 USA; 3 Department of Pathology, Harvard Medical School, Shattuck Street, Boston, MA 02115 USA; 4 Department of Genome Sciences, University of Washington, 15th Avenue NE, Seattle, WA 98195-5065 USA; 5 Canadian Institute for Advanced Research, University Avenue, Toronto, ON M5G 1M1 Canada; 6 Department of Bioengineering, University of Washington, 15th Avenue NE, Seattle, WA 98195-5061 USA

In the past 10 years, we have seen major advances in our ability to read human genomic DNA and detect variation. The variants we find have the potential to improve the diagnosis and treatment of human disease and also to define our unique traits. Although slower to catch up, we are now seeing equally rapid advances in the strategies used to interpret these variants in both coding and non-coding regions. Setting up a robust infrastructure, in terms of sequencing technology, pipelines for detection of all clinically significant variation, and analysis tools that incorporate the most effective approaches to variant interpretation, will be critical in delivering widespread and meaningful advances in patient care and in ensuring the accurate and informative application of genomic technology to healthcare.

## Toward comprehensive variant detection

Many platforms have been developed to detect different types of DNA variants in the germline and in the context of somatic cancer and mosaicism. For example, short read, next generation sequencing is routinely employed to detect short sequence variants, whereas Sanger sequencing is still used to confirm many variants. Karyotyping and chromosomal microarrays are platforms that are commonly used to detect structural variants. In addition, a myriad of other platforms and assays are used to detect partial gene deletions and duplications, common translocations, repeat expansions, and gene amplifications and to discern variation in homologous regions. Yet, maintaining these many platforms to detect the multitude of human variation is complex, costly, and difficult for laboratories, clinicians, and patients to navigate.

In this special issue of *Genome Medicine*, Lindstrand and colleagues [1] demonstrate the ability of whole genome sequencing to consolidate many of these platforms into a single approach for detecting a wide range of human variation types. The next step will be to democratize the computational tools needed to identify and annotate the different types of variation accurately, so that every laboratory that can generate a whole human genome sequence will be capable of highly sensitive and specific detection of all types of human genomic variation that have clinical consequences.

## Tools needed to support comprehensive variant interpretation

Although the detection of human genetic variation is a necessary first step, many resources are needed to support the accurate interpretation of the identified variation. The human population is genetically diverse, both in the spectrum of benign variation and in variation implicated in disease. In this issue, Abul-Husn and colleagues [2] report an increased rate of variants of uncertain significance in non-European populations compared to European ones, particularly in populations with a higher proportion of African ancestry. This burden of variants of uncertain significance results from a lack of recruitment from underrepresented populations, which has created a paucity of knowledge of disease causality in these populations. Diverse cohorts of affected individuals in disease studies are therefore needed to build knowledge of genetic disease etiologies across all populations and to ensure equitable benefit to all individuals from genomic medicine. The findings reported by Abul-Husn and colleagues [2] also highlight how large and diverse catalogs of human genetic variation across geographical populations are critical for ruling out the possibility that variants that are rare in one population but commonly observed in another are disease causing.

Also critical for variant interpretation are rigorous approaches for assessing the diversity of functional assays that are used to discern which variants disrupt the function of a gene product and which do not. This task is difficult because most gene products have a plethora of functions, sometimes in diverse cell types or even in an organismal context. In this special issue, Brnich and colleagues [3] propose a rigorous strategy to ensure that functional assays are well-validated before the data they generate are applied to routine clinical interpretation of variants. These recommendations have been developed for the evaluation and application of functional evidence within the ACMG/AMP variant interpretation framework [4], and are a key step forward in reducing discordance in the application of evidence codes.

Furthermore, once a functional assay has been validated, it can be multiplexed to enable comprehensive assessment of the effects of one or more classes of variation, thereby enabling streamlined and accurate genetic interpretation. Multiplexed functional assays are particularly useful for assessing classes of variation that are difficult to interpret, such as missense and splice site variation. Although promising, multiplexed functional assays present a set of unique challenges for both the researchers that develop them and the clinicians using the functional data they produce. Thus, Gelman and colleagues [5] make recommendations for how the developers of multiplexed functional assays should evaluate assay performance and report assay results. They also provide guidance to clinicians on how the quality and clinical utility of large-scale functional datasets can be evaluated, and on how these data can be incorporated into routine variant interpretation.

## Novel approaches to the identification of candidate disease loci

Traditional approaches to identify the genetic causes of rare disease continue to yield novel gene discoveries, including aggregating cases with extremely rare, highly penetrant phenotypes that share common disrupted candidate genes. Nevertheless, other human diseases have been harder to tackle because they are defined by nonspecific phenotypes or because they arise from variants at multiple loci. Examples include autism and congenital heart disease. However, with the ability to sequence both disease and control cohorts of individuals at scale, including trios that enable the detection of de novo variation, statistical frameworks are now able to highlight candidate disease loci with increasing precision. Lal and colleagues describe combined de novo burden analysis with grouping of paralogous genes to enable the identification of 28 strong candidate genes for neurodevelopmental disorders. Notably, these candidates are expressed in the brain and exhibit evolutionary constraint [6]. Another challenge is the interpretation of balanced structural variation, where possible drivers of pathogenicity are difficult to identify. Using a combination of experimental and computational approaches examining both direct disruption and indirect, chromatin-mediated effects, Middelkamp and colleagues [7] prioritized causal genes for previously uninterpretable de novo structural variants that were identified in the context of congenital abnormality or intellectual disability. In summary, the large scale aggregation of well-phenotyped individuals with diseases, through data sharing programs and the application of innovative methods of analysis, we will eventually build a comprehensive understanding of the genes and genomic regions that contribute to human disease.

## Supporting somatic variant interpretation in cancer

The interpretation of rare disease genetic variation has been hugely aided by systematic guidance [4] and by the routine sharing of variant interpretations in ClinVar. More recently, guidelines have been released to provide initial guidance for the interpretation of somatic variants, taking into account the added complexity of multiple dimensions of clinical relevance, including diagnosis, prognosis, and drug responsiveness [8]. These guidelines have better enabled the cancer community to standardize cancer variant assessment and to build shared community resources. These improvements are critical because they can empower the rapidly growing application of genetic testing in cancers, the results of which are critical to accurate prognosis and treatment guidance. In this issue, Lever and colleagues [9] demonstrate a text-mining approach to gather data from the literature on thousands of biomarkers and to deposit the information in a publicly accessible database called CIViCmine. He and colleagues [10] apply computational approaches to consume pre-annotated files and to apply criteria for clinical assessment. Both approaches enable the prioritization of variants identified in tumors for further review. Furthermore, Danos and colleagues [11] describe improvements to CIViC, which is an open platform for community curation of somatic variation. These improvements, which include common data models and standard operating procedures, are designed to support consistent and accurate interpretation of variants in cancer.

## Conclusion and future directions

As genomic medicine success stories continue to appear, we will confront an ever-growing number of genomes to analyze and genetic variants to interpret. Both tasks are difficult because of the complexity of the human genome and its diversity of variants, as well as the challenge of amassing sufficient data to interpret variants. This special issue describes some of the advances in variant detection, scaling of experiments, improvements in computational approaches, and construction of community resources that are helping to confront these challenges. Although this progress is promising, more work is needed. For example, we must develop an inexpensive, widely deployed pipeline for assembling whole genome sequences and detecting variants. We must apply such a pipeline to diverse human populations, at scale, in order to understand the true extent of common genetic variation. We must deploy multiplexed functional assays to quantify the effect of variation at many, if not most, disease-associated loci. Finally, we must unite these resources by adopting a coherent set of standards and a rigorous culture of data sharing. If successful, we will enable all individuals to benefit from the routine application of genomics to both disease diagnosis and genome-enabled disease prevention.

## References

[1] Lindstrand A, Eisfeldt J, Pettersson M, Carvalho CMB, Kvarnung M, Grigelioniene G (2019). From cytogenetics to cytogenomics: whole-genome sequencing as a first-line test comprehensively captures the diverse spectrum of disease-causing genetic variation underlying intellectual disability. Genome Med.

[2] Abul-Husn NS, Soper ER, Odgis JA, Cullina S, Bobo D, Moscati A, et al. Exome sequencing reveals a high prevalence of BRCA1 and BRCA2 founder variants in a diverse population-based biobank. Genome Med. 2019. 10.1186/s13073-019-0691-1 10.1186/s13073-019-0691-1 PMC6938627 31892343

[3] Brnich SA, Abou Tayoun AN, Couch FJ, Cutting G, Greenblatt MS, Heinen CD, et al. Recommendations for application of the functional evidence PS3/BS3 criterion using the ACMG/AMP sequence variant interpretation framework. Genome Med. 2019. 10.1186/s13073-019-0690-2 10.1186/s13073-019-0690-2 PMC6938631 31892348

[4] Richards S, Aziz N, Bale S, Bick D, Das S, Gastier-Foster J (2015). Standards and guidelines for the interpretation of sequence variants: a joint consensus recommendation of the American College of Medical Genetics and Genomics and the Association for Molecular Pathology. Genet Med.

[5] Gelman et al. Recommendations for the collection and use of multiplexed functional data for clinical variant interpretation. Genome Med. 2019. 10.1186/s13073-019-0698-7 10.1186/s13073-019-0698-7 PMC6925490 31862013

[6] Lal D, May P, Samocha KE, Kosmicki JA, Robinson EB, MØller RS, et al. Gene family information facilitates variant interpretation and identification of disease-associated genes. bioRxiv 159780; 10.1101/159780 10.1186/s13073-020-00725-6 PMC7079346 32183904

[7] Middelkamp S, Vlaar JM, Giltay J, Korzelius J, Besselink N, Boymans S, et al. Prioritization of genes driving congenital phenotypes of patients with de novo structural variants. Genome Med. 2019. 10.1186/s13073-019-0692-0 10.1186/s13073-019-0692-0 PMC6894143 31801603

[8] Li MM, Datto M, Duncavage EJ, Kulkarni S, Lindeman NI, Roy S (2017). Standards and guidelines for the interpretation and reporting of sequence variants in cancer: a joint consensus recommendation of the Association for Molecular Pathology, American Society of Clinical Oncology, and College of American Pathologists. J Mol Diagn.

[9] Lever J, Jones MR, Danos AM, Krysiak K, Bonakdar M, Grewal J, et al. Text-mining clinically relevant cancer biomarkers for curation into the CIViC database. Genome Med. 2019. 10.1186/s13073-019-0686-y 10.1186/s13073-019-0686-y PMC6891984 31796060

[10] He MM, Li Q, Yan M, Cao H, Hu Y, He KY (2019). Variant interpretation for Cancer (VIC): a computational tool for assessing clinical impacts of somatic variants. Genome Med.

[11] Danos et al. Standard operating procedure for curation and clinical interpretation of variants in cancer. Genome Med. 2019;11:76. 10.1186/s13073-019-0687-x 10.1186/s13073-019-0687-x PMC6883603 31779674

